# The microbial environment modulates non-genetic maternal effects on egg immunity

**DOI:** 10.1186/s42523-022-00195-8

**Published:** 2022-07-28

**Authors:** H. Pieter J. van Veelen, Joana Falcão Salles, Kevin D. Matson, G. Sander van Doorn, Marco van der Velde, B. Irene Tieleman

**Affiliations:** 1 Groningen Institute for Evolutionary Life Sciences, University of Groningen, P.O. Box 11103, 9700 CC Groningen, The Netherlands; 2 Wetsus, European Centre of Excellence for Sustainable Water Technology, P.O. Box 1113, 8900 CC Leeuwarden, The Netherlands; 3 Wildlife Ecology and Conservation Group, Wageningen University and Research, Droevendaalsesteeg 3a, 6708 PB Wageningen, The Netherlands

**Keywords:** Bird microbiota, Host–microbial interactions, Immune function, Maternal effect, Microbial environment

## Abstract

**Background:**

In a diverse microbial world immune function of animals is essential. Diverse microbial environments may contribute to extensive variation in immunological phenotypes of vertebrates, among and within species and individuals. As maternal effects benefit offspring development and survival, whether females use cues about their microbial environment to prime offspring immune function is unclear. To provide microbial environmental context to maternal effects, we asked if the bacterial diversity of the living environment of female zebra finches *Taeniopygia guttata* shapes maternal effects on egg immune function. We manipulated environmental bacterial diversity of birds and tested if females increased immunological investment in eggs in an environment with high bacterial diversity (untreated soil) versus low (gamma-sterilized soil). We quantified lysozyme and ovotransferrin in egg albumen and IgY in egg yolk and in female blood, and we used 16S rRNA gene sequencing to profile maternal cloacal and eggshell microbiotas.

**Results:**

We found a maternal effect on egg IgY concentration that reflected environmental microbial diversity: females who experienced high diversity deposited more IgY in their eggs, but only if maternal plasma IgY levels were relatively high. We found no effects on lysozyme and ovotransferrin concentrations in albumen. Moreover, we uncovered that variation in egg immune traits could be significantly attributed to differences among females: for IgY concentration in yolk repeatability *R* = 0.80; for lysozyme concentration in albumen *R* = 0.27. Furthermore, a partial least squares path model (PLS-PM) linking immune parameters of females and eggs, which included maternal and eggshell microbiota structures and female body condition, recapitulated the treatment-dependent yolk IgY response. The PLS-PM additionally suggested that the microbiota and physical condition of females contributed to shaping maternal effects on egg immune function, and that (non-specific) innate egg immunity was prioritized in the environment with low bacterial diversity.

**Conclusions:**

The microbial environment of birds can shape maternal effects on egg immune function. Since immunological priming of eggs benefits offspring, we highlight that non-genetic maternal effects on yolk IgY levels based on cues from the parental microbial environment may prove important for offspring to thrive in the microbial environment that they are expected to face.

**Supplementary Information:**

The online version contains supplementary material available at 10.1186/s42523-022-00195-8.

## Introduction

Immune function maturation depends on antigenic stimulation from the environment, which is a central process in shaping the immunological phenotype over the course of an individual’s life [1–3]. The immunological phenotype of a female, accumulated during her life, can potentially drive a phenotypically plastic component of her investment into offspring. This maternal immunity investment provides direct protection and primes the development of early- and late-life immunological phenotypes of offspring [4–6]. Such environment-dependent maternal influences that causally affect development and survival of offspring are referred to as non-genetic maternal effects [7–10], and can be ecologically and evolutionarily significant [8, 11, 12]. Microbial communities in an animal’s surroundings are a ubiquitous and rich source of antigens, and could thus be environmental drivers of maternal priming of offspring immunity.

Consistent with the fact that immunogens are stimulatory agents of an animals’ immune system, we previously reported experimental evidence that bacterial diversity in the environment can shape immune function on short time scales [13]. Whether these immunomodulatory effects of bacterial diversity cascade to immunological phenotypes of offspring through prenatal maternal effects has not been addressed. Thus far, immunological priming through maternal effects has been linked to other factors, such as resource limitation and postnatal parental care [14–16], as well as epigenetic inheritance [17]. These factors have been identified by challenging females with one or more immunogens, followed by quantification of immune traits of offspring [14], with particular focus on pathogens [e.g., 18]. However, to investigate the influence of bacterial diversity more broadly requires a different approach, because animals typically encounter diverse bacterial communities that vary in composition through space and time.

Experiments that manipulate the microbial environment of animals are needed to fully understand causal mechanisms driving maternal immune investment. Such an approach would also incorporate numerous other (non-pathogenic) microorganisms that trigger antibody production via a B-cell response [19–21]. Experimental evidence suggests that bacterial load (i.e. total bacterial abundance) has been linked to maternal immunological priming. For example, experimental reduction of bacterial load in nests lowered yolk carotenoid concentration in great tits *Parus major* and barn swallows *Hirundo rustica* [22, 23], and bacterial density on feathers predicted preen gland size and the composition of preen oil antimicrobials of great tits [24]. These findings suggested that environmental microbes can affect immunological priming, and alter immune function at short time scales, but did not implicate bacterial diversity. A basic understanding of whether environmental bacterial diversity affects immune investment requires explicit manipulation of bacterial diversity in an animal’s environment, followed by quantification of (transgenerational) immune function [20, 25, 26].

**Fig. 1:**
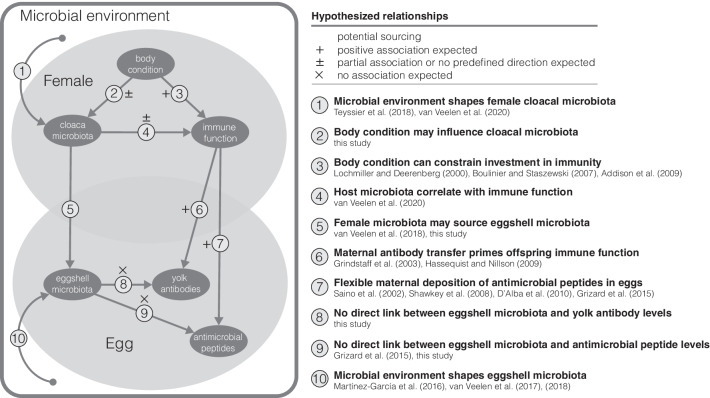
Conceptual model describing potential microbial environment effects on maternal immunological priming of avian eggs [9, 13–15, 27–36]

Prior work on adult zebra finches *Taeniopygia guttata* suggested that bacterial communities in the environment have immunomodulatory effects [13], but it remained unclear whether the degree of offspring priming associated with environmental bacterial diversity experienced by females. Here, we use the same study system to investigate if the diversity of the bacterial environment consequently shaped maternal immunological priming as non-genetic maternal effect. As the maternal microbiota can show signatures of the microbial environment [13, 27], we hypothesized that the diversity and composition of environmental bacterial communities shape non-genetic maternal effects on offspring immune function. In this study, we experimentally created two levels of environmental bacterial diversity and investigated their effects on maternal immune investment. Our first objective was to test if biomarkers of innate and adaptive immunity of eggs (i.e., antimicrobial peptides in egg albumen and IgY in yolk) were affected. Our second objective was to investigate transmission of maternal IgY by linking IgY concentrations in blood plasma and egg yolk. Our third objective was to explore relationships among maternal and egg immune function and cloacal and eggshell microbiota, and whether such relationships differed between experimental microbial environments. We illustrated potential associations among the components in a conceptual model (Fig. 1). We used partial least squares path modeling (PLS-PM) to explore the direct and indirect relationships among the immune biomarkers, cloacal microbiota, and body condition of the female, and immune biomarkers and shell microbiota of eggs. We predicted that environmental bacterial diversity influences maternal immune investment in eggs, and we predicted positive relationships between maternal and egg immune function. Ultimately, we expected egg immunity to be contingent on the structure of the maternal microbiota (as a maternal effect) but not the eggshell microbiota (as a direct environmental microbial effect).

## Methods

### Ethics statement

This study was carried out obeying the Dutch Law on animal experimentation under licence DEC6314A of the Institutional Animal Care and Use Committee of the University of Groningen.

### Experimental design and sample collection

#### Experimental treatment

We divided commercially acquired soil (clay ~ 40%, sand ~ 40%, organic matter ~ 20%) in two batches and applied three cycles of 25 kGy gamma irradiation (Synergy Health Ede B.V, the Netherlands) to one batch, creating a highly reduced microbial environment, hereafter referred to as the ‘low diversity’ soil treatment (Additional file 1: Fig. S1). We used the second soil batch as a high diversity microbial environment, hereafter referred to as the ‘high diversity’ soil treatment. We applied either high or low diversity soil as a ~ 2-cm deep bedding layer in cages (50 × 50 × 40 cm) housing zebra finches *Taeniopygia guttata*. Birds originated from a long-term in-house breeding population. At the start of the experiment birds were 1.5 years of age, except two individuals of 3.5 years. We maintained experimental microbial environments through biweekly cleaning of bedding trays, followed by sterilisation (70% ethanol) and replenishing with fresh low or high diversity soils (mean ± SEM: 15 ± 1 days, n = 4). We maintained soil moisture content by spraying daily with ~ 30 ml autoclaved water per cage, which corresponded to the daily water loss (unpublished data). We analysed temporal patterns of soil bacterial community structure by sampling soil from cages three times between each replacement (at day 3, 10, 14). These analyses demonstrated that experimental soil diversity and composition remained stable over 2-week periods (Additional file 1: Fig. S1) [13].

#### Species, housing and experimental timeline

To experimentally test if females adjust investment in antimicrobial defenses of their eggs based on the microbial environment that they experience, we moved 53 adult female and 54 adult male zebra finches from single-sex outdoor aviaries to indoor cages. Birds were housed for 8 weeks in single-sex pairs to prevent breeding but to allow physiological acclimation to experimental microbial environments (ambient temperature at 20 °C  ± 1, relative humidity at 55% ± 15 and a 12:12 h light–dark (L:D) cycle). Birds were then randomly assigned to a treatment, to one of two replicate rooms, and to one of 12 single-sex cages (situated in a block of 3 × 4) per room. Up to three single-sex groups of surplus individuals were in the same rooms.

We fed birds with ad libitum gamma-irradiated (3 × 25 kGy) seed mixture and provided autoclave-sterilized water to limit potential dietary effects on the gut microbiota. The water was supplemented with multivitamin/amino acid solution (0.2 μm-pore filter-sterilized; final concentration 4 g·l^−1^, Omni-vit, Oropharma N.V., Belgium) to compensate potential vitamin degradation from seed irradiation. We thoroughly cleaned and sterilized (70% ethanol) water and food dispensers two times per week to reduce bacterial growth and its potential influence on the bird’s microbiota.

After 8 weeks of experimental conditions, we randomly paired males and females within each room, increased daytime (by 1 h per day to 16 h:8 h L:D), and supplied cages with sterilized (70% ethanol) plastic nest boxes (van Riel Distripet B.V., Waalwijk, the Netherlands) and autoclaved artificial nest material (Quiko GmbH, Bocholt, Germany) to stimulate breeding activity**.** Pairs with a single completed clutch were removed from the experiment after 14 weeks, or shortly after a female completed two clutches within that period.

#### Sample collection

##### Birds

We measured body mass using a sterilized digital balance and collected a ~ 150 μl blood sample and a cloacal sample using a sterile cotton swab (Vacutest Kima, Arzegrande, Italy) from each female (n = 45) after the breeding period (i.e., after one or two complete clutches). Baseline cloacal microbiota have been described in Van Veelen et al*.* [13]. We did not collect cloacal swabs during the egg laying phase in order to reduce disturbance and the risk of ceasing egg laying before a clutch was complete. We used new pairs of nitrile gloves upon entering every room when handling experimental equipment or soils, and we used new gloves to handle birds, which we sterilized with 70% ethanol between individuals. We collected cloacal swabs in sterile 2-ml screw-cap vials that were kept on ice. Samples were stored at -20 °C immediately after all birds had been sampled.

##### Eggs

We aseptically collected and stored eggs individually in sterile plastic bags (Whirl–Pak®, Nasco, Fort Atkinson, WI, USA), which were secured in sterile 50 ml tubes. We stored eggs immediately at − 20 °C. All removed eggs were replaced with ethanol-sterilized plastic dummy eggs to encourage clutch completion and incubation. We marked the blunt end of first and second eggs of each clutch with a water-resistant marker to collect them when the first egg had been in the nest for 7 days. The first two eggs of each clutch were left in the nest to be incubated and intended for a separate study, but we included 20 of these eggs without embryos (Additional file 1: Table S1) to the analyses of egg immune defenses reported here. Subsequent eggs in the clutches (i.e., third to sixth egg of the laying sequence) were collected in the morning of the day they were laid. We collected a total of 262 eggs from first and second clutches (clutch size range: 3–6 eggs; see Additional file 1: Table S1 for a detailed overview of the collected and analyzed eggs).

### Laboratory analysis of immune function in egg albumen, yolk and female blood plasma

We dissected the eggs during the thawing process separating eggshells, albumen and yolk following Grizard et al. [37]. To remove residual albumen from the yolk sacks, we gently rolled thawing yolks on clean tissue before storing. We quantified lysozyme and ovotransferrin concentrations in egg albumen in duplicate following Horrocks et al*.* [38] and Horrocks et al*.* [39], respectively, using 10 μl albumen per sample per analysis. We measured albumen pH using a digital pH meter (Jenco Instruments, San Diego, CA). We quantified IgY concentrations in egg yolk (25 mg yolk homogenized in 400 µl 0.1% milk solution) in duplicate using enzyme-linked immunosorbent assays (ELISAs) following Grindstaff et al*.* [40] and Demas and Nelson [41], using an adjusted protocol described in [13]. We quantified haemagglutination and haemolysis titers in blood plasma following Matson et al*.* [42] and haptoglobin concentration in plasma following Matson et al*.* [43]. We reported averaged values of duplicate measurements for lysozyme, ovotransferrin, and IgY concentrations.

### DNA extraction and 16S rRNA gene amplicon sequencing

We ground eggshells in liquid nitrogen using sterile mortar and pestle for DNA extraction following Grizard et al*.* [37]. We prepared cloacal swabs for DNA extraction by aseptically removing the stalk from the swab fibers and transferred the fibers per sample in extraction tubes. We then extracted DNA from ~ 100 mg ground eggshell, 250 mg of homogenized composite soil samples, and from cloacal swab fibers using the MoBio PowerSoil DNA isolation kit (MoBio laboratories, Carlsbad, CA, USA) following the manufacturer’s protocol with an additional step: 0.25 g of 0.1 mm zirconia beads was used in three 60 s cycles of bead beating (beads and Mini-bead beater, BioSpec Products, Bartlesville, OK, USA) to enhance mechanical cell disruption. The V4/V5 region of the 16S rRNA gene was amplified in triplicate using primers 515F and 926R at Argonne National Laboratory, IL, USA, according to the Earth Microbiome Project protocol [44]. Amplification was followed by library preparation of pooled triplicates and 2 × 250 bp paired-end sequencing using V2 chemistry on an Illumina MiSeq. The sequencing runs included 22 technical negative extraction controls to test for kit contamination [45]. The negative controls covered every extraction kit that was used and included blank extractions and extractions with sterile swabs with and without zirconia beads.

### Sequence processing and assembly of amplicon sequence variants

In contrast to traditional 97% operational taxonomic unit (OTU) approaches, amplicon sequence variants (ASVs) lead to fewer false-positive taxon inferences while accurately illuminating cryptic diversity [46]. Hence, we quality-filtered and assembled sequences into error-corrected ASVs representing unique bacterial taxa using DADA2 v1.6.0 [46]. In total, we profiled 245 eggshell (excluding 20 eggshells with insufficient DNA content), 45 cloacal, and 69 soil bacterial communities, and assembled 9848 ASVs across these samples. We then assigned taxonomy to assembled ASVs using the Ribosomal Database Project (RDP) naïve Bayesian classifier implementation in DADA2 and the “RDP training set 16” and “RDP species assignment set 16” [47]. As implementations in QIIME2 [48], we used MAFFT to align ASV sequences [49] and FastTree2 to build a maximum-likelihood phylogenetic tree [50]. We then used *phyloseq* [51] to remove ASVs assigned to Archaea, chloroplasts, or mitochondria and ASVs without a bacterial phylum assignment. Two out of 22 negative controls produced amplicons (NC1 and NC8) but with distinctly lower read numbers compared with samples after quality filtering. Because of the low read counts in only two negative controls, we did not remove any ASVs from the sample data set prior to subsequent analyses.

Data sets were filtered prior to data analysis. Based on substantial variation in the coverage distributions of each sample type, which included several low coverage samples, we selected the top 80% of the samples from eggshells (n = 198; new median coverage = 3101 reads per sample; range = 339–24,815), the top 90% of the samples from cloacal swabs (n = 40; new median coverage = 4360; range = 726–78,049) and 100% of samples from soil (n = 69; median = 7138; range = 717–21,700). The remaining data comprised 7700 ASVs, which we used as input for beta-diversity analyses [52, 53]. Median sample coverage differed maximally 2.3 times between sample types (*χ*^2^ = 39.9, *df* = 2, *P* < 0.001), which is acceptable [52] for application of a variance-stabilizing transformation of the feature table using *DESeq2* [52–54] before calculating unweighted and weighted UniFrac as measures of phylogenetic beta-diversity [55].

### Statistical analyses

All statistical analyses were performed in R statistical software [56]. We used linear mixed models (LMMs) to test the effect of different microbial environments on egg immune indices [i.e., yolk IgY concentration (n = 154), albumen lysozyme (n = 139), and ovotransferrin concentrations (n = 119)]. By including female identity as random effect, we statistically accounted for non-independence of eggs sampled from the same female when evaluating the effect of treatment. We tested treatment effect by modelling microbial environment as a fixed factor, clutch number as categorical confounding factor, egg sequence as ordinal covariate, and replicate room as additional random effect. Since albumen pH can influence lysozyme and ovotransferrin activity [35], we included albumen pH as additional covariate in LMMs for these antimicrobial compounds. We performed a log-transformation of lysozyme concentration to meet the assumptions for residual normality and homoscedasticity. We performed ANOVAs using *lme4* and *lmerTest* [57, 58], and then extracted model predictions using *effects* [59]. To test if maternal investment consistently differed among females, we calculated within-female repeatabilities adjusted for fixed effects (*R*_adj_) from the LMMs for each measure using *rptR* [60, 61]. In addition, we summarized the variation of immune indices as a pairwise distance matrix among egg samples (referred to as ‘immune index’) using *vegan* [62] including those eggs that were fully analyzed for concentrations of lysozyme, ovotransferrin, and IgY (n = 115; Additional file 1: Table S1). We then used distance-based redundancy analysis (db-RDA) to test the effect of microbial environment on multitrait egg immunity while constraining ordination by clutch size, egg number, and female identity using the capscale function.

Furthermore, since IgY levels could be compared directly between females and their eggs for each female-egg dyad, we analyzed this relationship to compare and interpret maternal immunological priming in the two experimental microbial environments. We first tested if female plasma IgY concentrations differed between environments. To test this we used a LMM with experimental treatment as fixed factor and modelled random intercepts for female identity and replicate room. We then analysed the relationship between yolk IgY and plasma IgY concentrations using a similarly structured LMM with the additions of female plasma IgY concentration as fixed predictor of egg yolk IgY concentration and its interaction with treatment.

### Partial least squares path modelling (PLS-PM)

We used partial least squares path modelling (PLS-PM) to create a more holistic view of immune functions of females and eggs in the context of the microbial environment. PLS-PM is a statistical method that utilizes dimension reduction to allow analysis of a system of cause-effect relationships among blocks of (high dimensional) observational data [63]. Our goal here was to refine existing hypotheses and potentially to generate new hypotheses about the complex system of interactions between microbial and immunological components of mothers and eggs in the nest environment. The unidirectional paths that we included in the path model reflect hypothesised causal relationships from the ecological immunology framework (Fig. 1). Because PLS-PM is primarily for generating hypotheses, not for testing them, the method does not impose formal restrictions on data distributions. The method is particularly suited to integrate data reduction with path modelling approaches to identify and quantify direct and indirect relationships among multivariate data sets [e.g., 64, 65]. Hence, PLS-PM allowed for integration of maternal immune function and a body condition index (i.e., residual body mass after correcting for structural size using tarsus length), maternal cloacal microbiota (i.e., non-genetic maternal effects) and the eggshell microbiota (i.e., direct environmental effect) to explore if and how these components relate to egg immunity.

We selected empirical data as input for the path model (i.e., manifest variables) based on data completeness, pairwise collinearity among variables, and intrinsic structure of maternal and eggshell microbiota data. We simplified the microbiota data sets by selecting the largest clades identified by k-means clustering; maternal and eggshell microbiota each contained three distinct clusters. We used the clusters as formative indicators for latent variables representing the maternal and eggshell microbiotas. We excluded ovotransferrin concentration in albumen and lysis titer of blood plasma due to a lack of data and variance, respectively. We utilized data of 105 eggs (out of 198; 47 and 58 from high and low diversity treatments, respectively) from 29 birds for which quantitative measures of all maternal and egg parameters were available. Maternal immune function was defined by two latent variables: one for natural antibody-induced agglutination titer as a measure of constitutive innate immunity, and one comprising both IgY concentration and haptoglobin level because of their collinearity, which we referred to as maternal ‘immune index’. To ascertain that these indicators reflected the latent variable in the same direction, we inversed haptoglobin concentration. This adjustment enhanced the degree to which latent variables reflected the observed variables in the path model [63]. Because haptoglobin concentration signals the degree of inflammation, inversed lower values indicated more inflammation, which were together with high IgY levels predicted to reflect bacterial diversity.

Under the assumption that the hypothesised causal relationships between variables (i.e., the ‘structural model’) are correct, it is possible to explore within the PLS-PM framework whether two experimental groups differ in the strength of particular associations between groups of variables. Hence, to assess whether maternal effects differed between experimental microbial environments, we compared the path coefficients (i.e., standardized partial regression coefficients) of the structural model between treatment groups using bootstrap resampling (n = 1000) and a t-test based on the bootstrap standard errors [63]. Comparing between microbial environments, we interpreted significant differences (critical FDR-corrected *q* < 0.1) in the direction or strength of path coefficients between females and eggs as support for microbial environment-dependent maternal immune investment. Treatment-specific *t*-test results for the magnitude of path coefficients were extracted from the PLS-PM. Finally, we validated the robustness of path coefficients and coefficients of determination (R^2^) for different variants of the structural model using 1000 bootstraps for estimating 95% confidence intervals. We used the R package *plspm* to construct the path models [66]. Because of limited a priori understanding of causal links between microbiota and immune function, we remained cautious with inferring path coefficients as causal evidence and we avoided quantitative predictions. Instead, we limited the implications of PLS path model results to refine current hypotheses and for guiding new ideas about microbial environment-dependent maternal effects.

## Results

### No effect of environmental microbial diversity on egg immunity

**Fig. 2:**
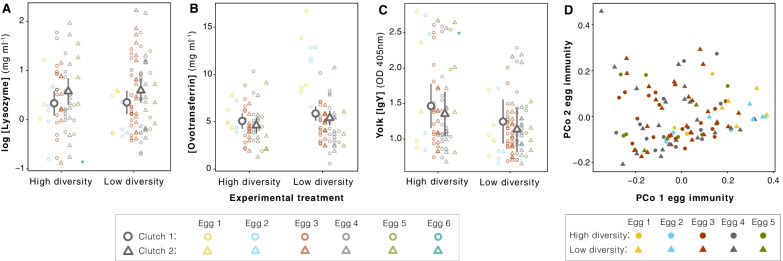
Experimental microbial environmental effects on egg immune function. **A** Lysozyme concentration (mg ml^−1^; log scale), **B** Ovotransferrin concentration (mg  ml^−1^), **C** IgY concentration in yolk (OD_405nm_), and **D** the first two principal coordinate axes of a multivariate immune index that represents the variation of the indices presented in **A**–**C**. Individual egg samples are presented by laying sequence (color) and stratified by clutch number (shape in **A**–**C**) or treatment (shape in **D**). None of the egg immune indices were significantly different between the two experimental microbial environments (Table 1)

Tests for overall experimental effects on the three egg immune parameters revealed no significant effect of experimental microbial environment on lysozyme and ovotransferrin concentrations in albumen and total IgY concentration in yolk of zebra finch eggs (Fig. 2A–C; Table 1). Multivariate analysis (distance-based RDA) of egg immune defense traits, which simultaneously considered the variation of the three egg immunity measures, also did not reveal clustering of zebra finch eggs by experimental group (Fig. 2 D; Table 1). Log-transformed lysozyme concentration was 0.24 mg ml^−1^ higher in second clutches compared to first clutches (*t* = 2.34, *df* = 125, *P* = 0.03; Table 1), and ovotransferrin decreased with 1.11 mg ml^−1^ per egg along the laying sequence (*t* = 4.46, df = 116, *P* < 0.001). Absorbance of antigen-specific IgY in second clutches was 0.11 units (OD_405_) lower than in first clutches (*t* = 3.85, *df* = 121, *P* < 0.001), but did not vary along the laying sequence (*t* = 1.59, df = 121, *P* = 0.11).

**Table 1:** Analysis of variance of egg immune function indices

Response	Fixed	*df*^a^	*F*	*P*
Albumen lysozyme log-scale (mg ml^−1^)	Experimental treatment	1, 33	0.01	0.908
	Clutch number	1, 116	4.90	**0.029**
	Egg sequence	1, 128	0.14	0.709
	pH	1, 122	0.62	0.432

	Random	Variance		
	Female identity	0.130		
	Replicate room	0.000		
	Residual	0.357		

	Fixed	*df*^a^	*F*	*P*
Albumen ovotransferrin (mg ml^−1^)	Experimental treatment	1, 114	2.93	0.090
	Clutch number	1, 114	1.08	0.301
	Egg sequence	1, 114	21.64	** < 0.001**
	pH	1, 122	0.24	0.627

	Random	Variance		
	Female identity	0.000		
	Replicate room	0.000		
	Residual	6.226		

	Fixed	*df*^a^	*F*	*P*
Yolk IgY concentration (absorbance)	Experimental treatment	1, 1.88	0.98	0.433
	Clutch number	1, 118	14.45	** < 0.001**
	Egg sequence	1, 119	2.62	0.108

	Random	Variance		
	Female identity	0.216		
	Replicate room	0.024		
	Residual	0.029		

Multivariate immune index (db-RDA)^b,c^		*df*^a^	*F*	*P*
	Experimental treatment	1, 103	1.36	0.196
	Clutch number	1, 103	1.37	0.195
	Egg sequence	1, 103	5.02	** < 0.001**

Bold values denote significant effects (alpha = 0.05)

^a^Denominator degrees of freedom based on Satterthwaite approximation

^b^Distance-based Redundancy Analysis based on a Bray–Curtis dissimilarity matrix of three immune indices

^c^Marginal effects estimated with permutations stratified by female identity

### Consistent differences in egg immunity at the level of the female

In contrast to group-level experimental effects, among-female repeatability was significant for lysozyme concentration, IgY concentration and the multivariate immune index, but not for ovotransferrin concentration (Table 2; Additional file 1: Fig. S2). These repeatabilities imply that immunological variation in eggs can be explained by consistently different transfer by females.

**Table 2:** Adjusted repeatabilities of egg innate immune function for individual female zebra finches

Immune index		*R*_adj_	SE	95% CI (lower, upper)	*P*
Albumen lysozyme					
log-scale (mg ml^−1^)		0.268	0.095	0.073, 0.442	**0.001**
Albumen ovotransferrin (mg ml^−1^)		0	0.052	0, 0.17	1.000
Yolk IgY concentration (OD_405nm_)		0.804	0.113	0.503, 0.923	**0.001**
Multivariate immune index	High diversity PCo 1	0.214	0.2	0, 0.568	0.131
	High diversity PCo 2	0.406	0.18	0, 0.701	**0.011**
	Low diversity PCo 1	0	0.068	0, 0.236	1.000
	Low diversity PCo 2	0.277	0.141	0, 0.549	**0.010**

Bold values denote significant effects (alpha = 0.05)

### Maternal transfer of total antigen-specific antibodies to eggs is conditional on the microbial environment and maternal antibody levels

**Fig. 3:**
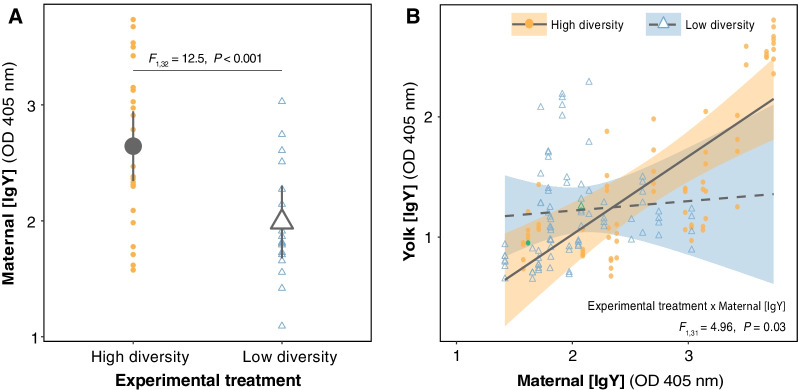
Experimental effect on the relationship between maternal and egg yolk IgY concentrations. **A** Elevated maternal IgY concentration in a high diversity microbial environment. **B** Egg IgY concentration increases with maternal IgY concentration only in the high diversity microbial environment. Lines depict linear mixed model predictions (± 95% CI)

To discern environmental microbial effects and effects of females, we first assessed whether the maternal plasma IgY levels differed between experimental treatments after egg laying had been completed. Maternal IgY concentration was higher in the high diversity microbial environment compared with low diversity microbial environment (*F*_1,32_ = 12.5, *P* = 0.001; Fig. 3A). We then analysed the relationship between maternal IgY concentration in blood plasma and in yolk of their eggs as a direct measure of maternal immunological priming. Utilizing maternal IgY concentration to predict egg yolk IgY concentration showed a significant interaction between experimental treatment and maternal IgY concentration (*F*_1,31_ = 4.96, *P* = 0.033; Fig. 3B). Egg yolk IgY concentration was positively associated with maternal IgY concentration in birds that experienced the high diversity microbial environment, but not in birds that experienced the low diversity microbial environment.

### The path model points out that maternal immunological priming of eggs may depend on the experienced microbial environment when females are in good body condition

**Fig. 4:**
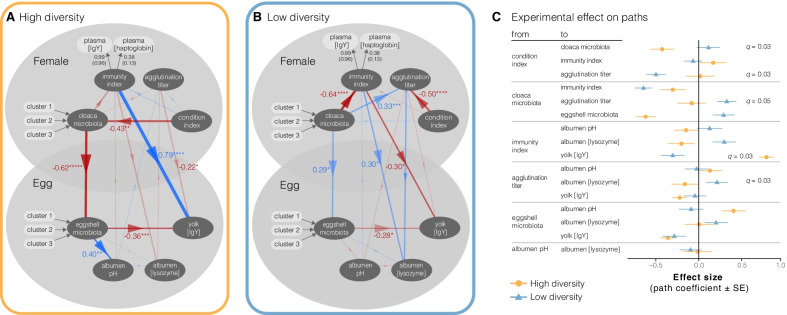
PLS-PM predictions link environmental and maternal microbiota to egg immune function. PLS-PM structural model representations. (**A**, **B**) depict predicted path coefficients that were extracted from the global model for experimental microbial environments with **A** High diversity and **B** Low diversity. **C** The experimental treatment effect on path coefficients was assessed with a bootstrap procedure and a *t*-test, where effects with FDR *q* < 0.1 were considered significant. **A**, **B** Dark grey ellipses depict (uni- or multivariate) latent variables and light grey rectangles represent manifest variables of either reflective or formative indicators of the latent variables. Colored arrows represent the path predictions (blue = positive; red = negative), line weight is proportional to the effect size (arrow labels); asterisks denote the probability that path coefficient is not zero: * *P* < 0.05, ** *P* < 0.01, *** *P* < 0.001, **** *P* < 0.0001

We evaluated our conceptual ideas (see Fig. 1) on how maternal immunological priming may depend on the microbial diversity in the offspring’s expected future environment using PLS-PM. The path model was indicative of strong and significant differences in maternal immunological priming of eggs between experimentally manipulated microbial environments that females (and their paired males) experienced in the 8 weeks prior to nesting and egg laying (Fig. 4 C). We presented more detailed summaries of treatment-specific path coefficients and bootstrap *t*-test results for experimental differences as Additional file 1: Tables S2 and S3, respectively.

The maternal immune index which included maternal IgY and haptoglobin levels strongly and positively predicted the IgY concentration of egg yolk (*r* = 0.79, *P* < 0.0001; Fig. 4A) of birds that experienced high diversity environmental microbial conditions, whereas it predicted an opposite association for birds that lived in the low diversity microbial environments (*r* = − 0.30, *P* = 0.032; Fig. 4B). Conversely, maternal innate immune function, measured as natural antibody-induced agglutination titer, negatively associated with the IgY conconcentraion in egg yolk in the high diversity environment (*r* = − 0.22, *P* = 0.015; Fig. 4A), whereas no association was predicted for the environment with low microbial diversity. The maternal immune index of females that experienced an environment with low microbial diversity positively predicted lysozyme concentration in egg albumen (*r* = 0.30, *P* = 0.034; Fig. 4B). The lysozyme concentration was not different in eggs between experimental microbial environments. Hence, the associations between maternal immune index and egg yolk IgY concentration (Fig. 4A), as well as the association between maternal agglutination titer and egg lysozyme concentration (Fig. 4B), significantly differed between microbial environments (Fig. 4 C; Additional file 1: Table S3). This suggests that females that experienced relatively high bacterial diversity in their environment invest in egg yolk IgY for their offspring rather than in non-specific innate defences, whereas under relatively low bacterial diversity the opposite is prioritized with increased lysozyme concentrations in albumen.

In our conceptual model, we included potential effects of general body condition (i.e. condition index defined as tarsus length-corrected mass) of females on maternal effects. Our path model revealed that the maternal condition index negatively predicted agglutination titer in the low diversity (*r* = − 0.50, *P* < 0.0001) but not in the high diversity microbial environment (*r* = 0.02, *P* = 0.92; Fig. 4C). An opposite pattern was observed for the relationship of body condition with cloacal microbiome structure (Fig. 4C), and no associations were found with the maternal immune index (Fig. 4A, B). Because the phylogenetic composition of the maternal microbiota did not differ between experimental treatment groups (weighted UniFrac: pseudo-*F*_1, 39_ = 0.03, *P* = 0.24; unweighted UniFrac: pseudo-*F*_1, 39_ = 0.03, *P* = 0.39; Additional file 1: Fig. S3) these results were based on the intrinsic structure in the cloacal microbiota (k-means clustering; k = 3). The path coefficients did not differ between treatments (Fig. 4C), likely because the variation among females within each treatment was considerable (Additional file 1: Fig. S3).

Maternal cloacal microbiota structure additionally associated with the maternal immune index of females (high diversity: − 0.30, *P* < 0.05; low diversity: − 0.64, *P* < 0.0001; Fig. 4A, B), but not differently between experimental microbial environments (Fig. 4C), and it associated with maternal agglutination titer only in the environment with low microbial diversity (Fig. 4B, C). These apparent associations between the maternal cloacal microbiota and the maternal immune index suggest within-individual processes linking the microbiota and immune function.

In addition, the structure of maternal and eggshell microbiotas were linked in both experimental microbial environments (Fig. 4A, B), but statistical support for an effect of experimental treatment was lacking (bootstrap *t* = 0.52, *df* = 103, *P* = 0.302; Fig. 4C). Eggshell microbiota predicted egg yolk IgY concentrations in both environments (Fig. 4A, B). The maternal immune index and agglutination titer were not associated with pH of egg albumen (Fig. 4A, B), whereas eggshell microbiota structure predicted albumen pH only in the high diversity environment (Fig. 4A). Albumen pH did not predict lysozyme concentration, which was in contrast with our expectations.

## Discussion

We have previously shown that environmental microbiomes can modulate immune responses in females [13]. Immunological differences due to environmental bacterial diversity, and, independent of that, consistent differences in maternal immune traits and cloacal microbiota features brought up the possibility that maternal immunological priming of offspring may be similarly affected. Our results revealed that the microbial environment and female traits interactively determined maternal immunological priming of eggs. Variation in albumen lysozyme, albumen ovotransferrin and yolk IgY, biomarkers of egg immune function, could not be independently explained by the microbial diversity of experimental environments alone. Instead, consistent differences among females formed an important source of variation of these biomarkers. The relationships between levels of immunoglobulin Y (IgY) in maternal plasma and egg yolk depended on microbial environment: only in the high diversity microbial environment females transferred more IgY to eggs when their plasma IgY levels were relatively high. Path modeling subsequently provided a systems-level perspective that recapitulated this latter pattern, and suggested that maternal cloacal microbiota and body condition contribute to shaping maternal effects on egg immunity. It additionally suggested that the agglutination titre of female blood plasma and lysozyme in egg albumen, both non-specific innate defenses, were prioritized in the environment with low microbial diversity. Few associations between bacterial diversity and immunity have been studied so far. Hence, we anticipate that our results, and a more general perspective on linking pressure posed by microbes to immune function, encourage further investigation of the role of microbial diversity – and its different components – on vertebrate immunological development within and across generations.

### Egg immune function

We found no independent effect of experimental microbial environment on levels of albumen lysozyme, albumen ovotransferrin and yolk IgY in eggs. Eggs varied markedly for all immune biomarkers and among-female repeatabilities for these biomarkers of egg immunity, up to 0.80 for egg yolk IgY, suggest that at least part of the immune variation among eggs could be attributed to differences among females. Since transfer of antibodies to egg yolk is associated to maternal plasma levels [15], and we previously found among-female repeatability of plasma IgY levels in these birds [13], our results comply with our expectation that among-female variation in IgY transfers to eggs. We found that a lesser degree of variation in lysozyme in albumen and IgY in yolk could be explained by clutch number, and of ovotransferrin in albumen by laying order. Effects of clutch number and laying order have been reported in other bird species, but their occurrence and directions can be species-specific and driven by other factors [e.g., 34, 35, 67, 68]. Differences in maternal transfer among females can arise due to both genetic and environmental factors [9, 15, 69]. We further discuss the environmental factors with a particular focus on the effects of the microbial component.

### Maternal antibody transfer: interacting effects of microbial environment and female

Assessing maternal transmission of IgY, we found that eggs contained the highest IgY levels in the high microbial diversity environment, but only in eggs produced by females with relatively high plasma IgY levels. This result supports our hypothesis that maternal antibody transfer to yolk is microbial environment-dependent, which indicates that the microbial environment may reorder priorities for maternal resource trade-offs. That would also suggest that transfer of maternal antibodies is not simply passive, which contrasts with earlier ideas [70, 71]. The consistent differences among females throughout the experiment raise the question which female traits influence maternal transfer. Body condition is a trait that in female King quails *Excalfactoria chinensis* has been shown to influence antibody transfer to eggs [72]. Factors implicated by other studies include energetic or nutritional budgets [16, 73–75] and age (reviewed in [76]). We supplied ad libitum sterilized food in our experiment, which makes resource balance an unlikely explanation for our findings. Likewise, age is an unlikely explanation, because the zebra finches in this study constituted a single captive cohort aged between 1 and 2 years. Based on the role of the microbial environment reported here, we propose that pressure posed by environmental microbial communities may reframe priorities for maternal investment tradeoffs when transfer of immunity becomes more important for offspring fitness.

### Path modeling: a systems-level perspective on maternal immune investment in eggs

We applied path modeling to explore maternal immune transfer in a systems-level perspective to identify unobserved relationships and indirect effects (see Fig. 1 for conceptual ideas), including associations among immune biomarkers, and data on cloacal microbiota and body condition as additional maternal traits. We caution that model results are based on the assumption that the structure of proposed relationships is correct. The model results suggested that relationships among maternal and egg immune parameters are microbial environment-specific. Particularly, the model fostered the hypothesis that adaptive immunity is prioritized by female zebra finches when they experience relatively high bacterial diversity, whereas innate defenses are prioritized under relatively low bacterial diversity. We propose that when microbial pressure is at least partly predictable, such as with annual or seasonal variations in environmental microbial communities [77, 78] or with diet-associated microbial communities [79, 80], phenotypically plastic immune investment could be expected. Furthermore, we propose that this plastic response may act on overall investment in immunity, as well as on the balance between innate and adaptive defenses, both of which may subsequently translate into non-genetic maternal effects.

Moreover, the path model brought forward the hypothesis that the maternal cloacal microbiota and body condition may contribute to shaping maternal effects on immunity. Based on these outcomes, we suggest that balancing maternal investment in innate and adaptive immunity may depend on sequential effects of the experienced microbial environment through alteration of the maternal microbiota as a sensor for microbial pressure. Future challenges remain to discern relative contributions of these different factors on phenotypically plastic responses of females, and how they interact to shape maternal effects on immunity.

### Conclusion and outlook: Non-genetic maternal effects on immune function in the context of microbial pressure

Our results constitute evidence of a direct link between bacterial diversity and female traits that interactively modulate egg immune function as maternal effects. These results offer further prospects for manipulation of microbial pressure to unravel how microbial diversity shapes short term and life-long effects on health and survival through non-genetic maternal effects. Furthermore, microbial load likely also contributes to microbial pressure by influencing the probability with which antigenic stimulation is prompted [81, 82]. We postulate that microbial pressure effectively triggers immune systems as a function of microbial diversity and load, each of which may or may not independently influence investment in immunity and the tradeoffs between adaptive and innate defenses. We suggest that ecological immunology could greatly benefit from a framework to quantify relative influences of microbial diversity, load, and their predictability, and by integrating this knowledge to predict their relative importance for investment in immune defenses.

## Supplementary Information

**Additional file 1.** Supplementary figures and tables.

## Data Availability

Sequence data are available in the European Nucleotide Archive under project accession numbers PRJEB45297 (cloacal samples), PRJEB30563 (soil samples) and PRJEB45531 (eggshell samples). All underlying metadata, immune function data and R scripts are available from GitHub: https://github.com/pietervanveelen/Zebra_finch_maternal_effect_immunity.
