# Supplementary material for: The microbial environment modulates non-genetic maternal effects on egg immunity
Source: Anim Microbiome. 2022 Jul 28;4:44. doi: 10.1186/s42523-022-00195-8 (PMC9331593; doi:10.1186/s42523-022-00195-8)
Supplement: Supplementary file 1 — Additional file 1. Supplementary figures and tables. [file 42523_2022_195_MOESM1_ESM.docx]

**Supplementary Figures and Tables**

This additional information accompanies:

**The microbial environment modulates non-genetic maternal effects on immunity**

H. Pieter J. van Veelen^1,2 *^, Joana Falcão Salles^1^, Kevin D. Matson^3^, G. Sander van Doorn^1^, Marco van der Velde^1^, B. Irene Tieleman^1^

^1^ Groningen Institute for Evolutionary Life Sciences, University of Groningen,

P.O. box 11103, 9700 CC, Groningen, The Netherlands

^2^ Wetsus, European Centre of Excellence for Sustainable Water Technology,

P.O. box 1113, 8900 CC, Leeuwarden, The Netherlands

^3^ Wildlife Ecology and Conservation Group, Wageningen University & Research, Droevendaalsesteeg 3a, 6708PB Wageningen, Netherlands

* Corresponding author:

H. Pieter J. van Veelen

Wetsus, European Centre of Excellence for Sustainable Water Technology,

Oostergoweg 9, 9811 MA, Leeuwarden,

The Netherlands

Email: [pietervanveelen2@gmail.com](mailto:pietervanveelen2@gmail.com)

**
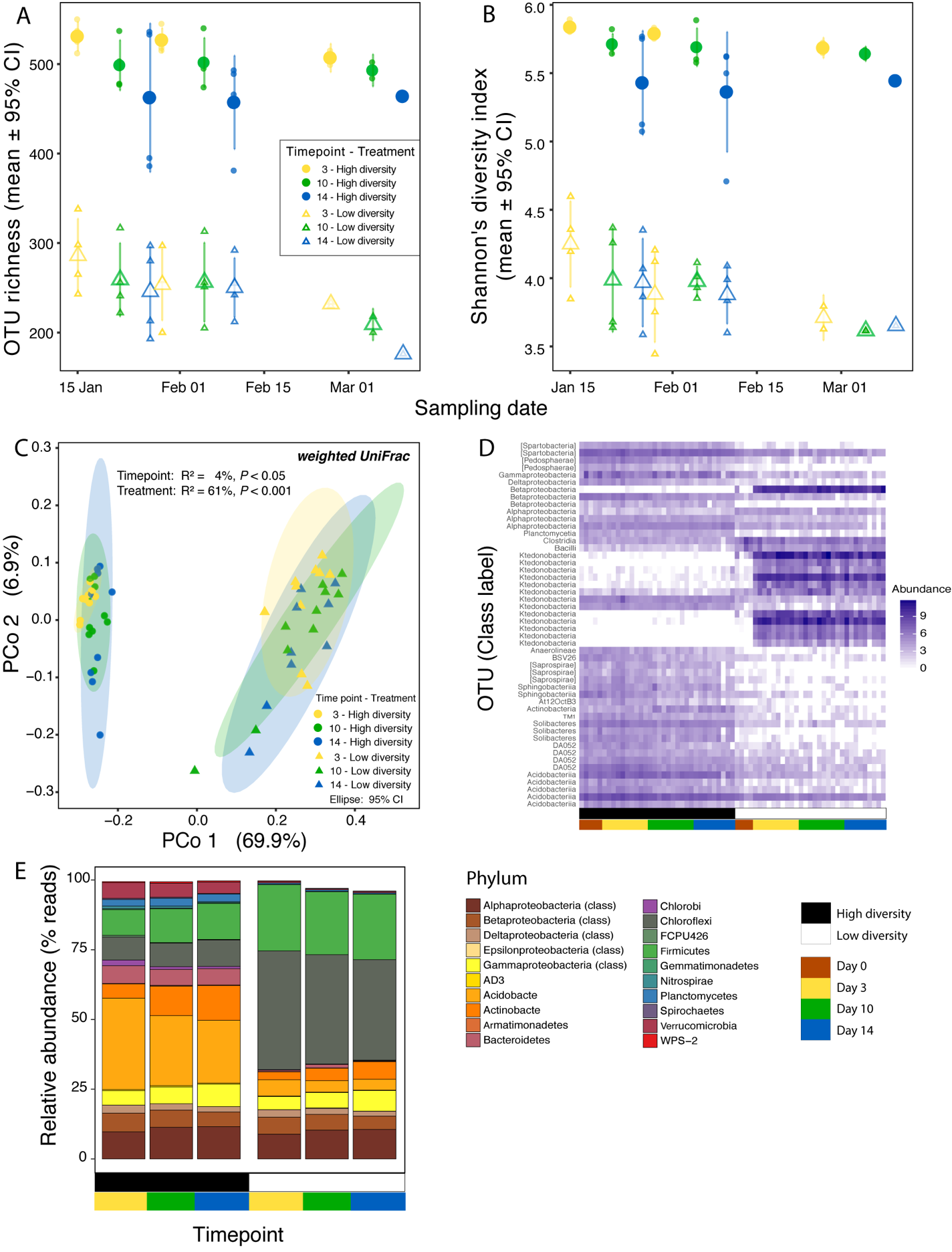
**

**Figure S1. Experimental soil bacterial community characteristics.** Relationship of a) OTU richness and b) Shannon diversity with sampling date during the experiment for each experimental soil treatment (closed circle = high diversity soil, open circle = low diversity soil) across time points between soil replacements (yellow = 3, green = 10 days, blue = 14 days). c) Principal coordinate analysis (PCoA) using weighted UniFrac distances of soil. d) Heatmap of soil samples showing variance-stabilised abundances of 50 most differentially abundant OTUs (labelled by bacterial class). e) Bacterial community structure represented by mean relative abundances of major bacterial groups (colours), stratified by time point for each of the two soil treatments. a) LMM OTU richness ANOVA; experimental treatment: *F*_1,54_ = 551.2, *P* < 0.001; time point: *F*_2,54_ =5.58, *P* < 0.01; b) LMM Shannon diversity ANOVA; experimental treatment: *F*_1,54_ = 600.7, *P* < 0.001; time point: *F*_2,54_ =4.08, *P* < 0.05. c) PERMANOVA: experimental treatment: Pseudo-*F*_1,54_ = 96.7, *P* < 0.001; time point: Pseudo-*F*_2,54_ =3.32, *P* < 0.05. This figure is reproduced from van Veelen et al. (2020).

**Figure S2**. Repeatability of egg immune function parameters. See Table 2 in the main text for details.


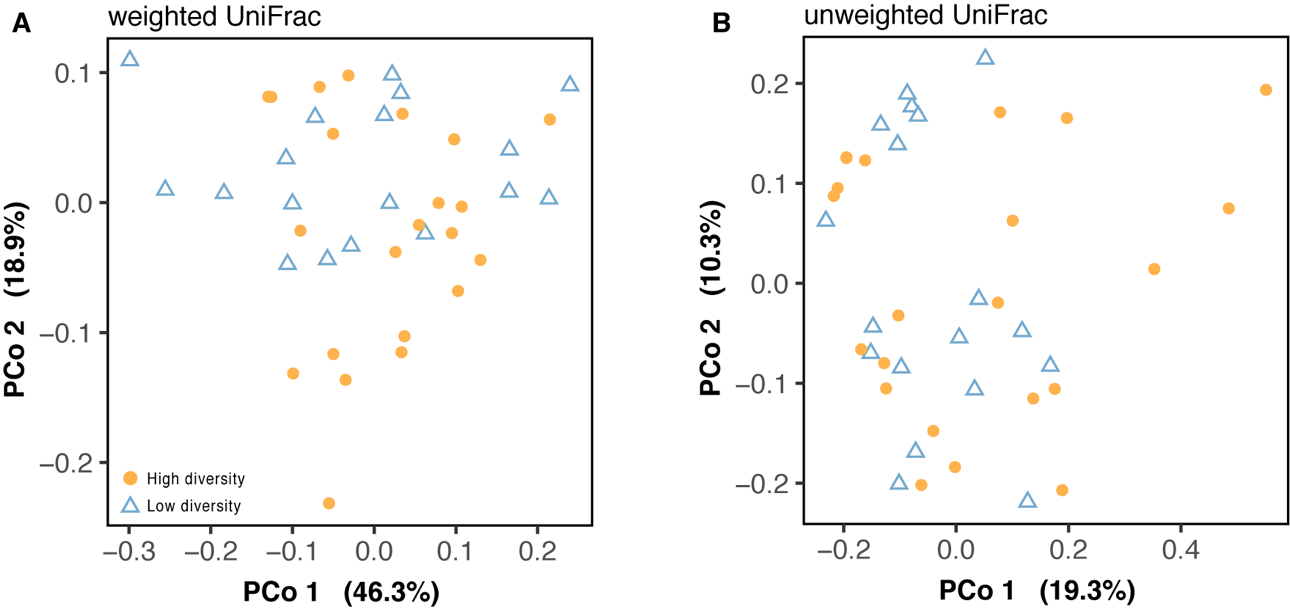


**Figure S3**. Phylogenetic composition of maternal microbiota in high and low diversity microbial environments. Post-laying cloacal microbiota compositions of female zebra finches as depicted by principal coordinates analysis. PERMANOVA analysis based on weighted (pseudo-*F*_1, 39_ = 0.03, *P* = 0.24) and unweighted UniFrac (pseudo-*F*_1, 39_ = 0.03, *P* = 0.39) indicated that cloacal microbiota compositions did not differ anymore between experimental microbial environments with high or low microbial diversities after egg laying, whereas the pre-laying period was characterized by different cloacal microbiota in high and low diversity environments treatments (van Veelen et al., 2020).

**Table S1.** Description of collected and analysed zebra finch eggs

|  |  | High diversity soil treatment | | | | |
| --- | --- | --- | --- | --- | --- | --- |
|  |  | n collected | n analysed | | | |
|  |  |  | **[lysozyme]** | **[ovotransferrin]** | **total [IgY]** | **All measures** |
| Clutch 1 | Egg 1 | 16 | 1 | 3 | 3 | 1 |
|  | Egg 2 | 17 | 4 | 3 | 3 | 3 |
|  | Egg 3 | 16 | 14 | 10 | 16 | 8 |
|  | Egg 4 | 13 | 10 | 8 | 13 | 6 |
|  | Egg 5 | 3 | 3 | 3 | 3 | 3 |
|  | Egg 6 | 1 | 1 |  | 1 |  |
| Clutch 2 | Egg 1 | 13 | 2 | 2 | 2 | 2 |
|  | Egg 2 | 13 | 1 | 0 | 1 |  |
|  | Egg 3 | 13 | 9 | 11 | 13 | 8 |
|  | Egg 4 | 13 | 11 | 9 | 13 | 8 |
|  | Egg 5 | 4 | 4 | 4 | 4 | 4 |
| subtotal |  | 122 | 60 | 53 | 72 | 43 |
|  |  | **Low diversity soil treatment** | | | | |
| Clutch 1 | Egg 1 | 18 | 3 | 3 | 3 | 3 |
|  | Egg 2 | 16 | 4 | 4 | 4 | 3 |
|  | Egg 3 | 17 | 17 | 14 | 17 | 16 |
|  | Egg 4 | 15 | 15 | 12 | 15 | 13 |
|  | Egg 5 | 4 | 4 | 2 | 4 | 3 |
|  | Egg 6 |  |  |  |  | 1 |
| Clutch 2 | Egg 1 | 16 | 2 | 2 | 2 | 2 |
|  | Egg 2 | 18 | 1 | 1 | 1 | 1 |
|  | Egg 3 | 17 | 17 | 14 | 17 | 13 |
|  | Egg 4 | 15 | 13 | 11 | 15 | 13 |
|  | Egg 5 | 4 | 3 | 3 | 4 | 4 |
| subtotal |  | 140 | 79 | 66 | 82 | 72 |
| total |  | **262** | **139** | **119** | **154** | **115** |

**Table S2.** Partial Least Squares path coefficients for associations between maternal and egg parameters per experimental microbial environment

|  | High diversity | | | | Low diversity | | | |
| --- | --- | --- | --- | --- | --- | --- | --- | --- |
|  | **cloacal microbiome** | | | | | | | |
| Intercept | 0.000 | 0.135 | 0.000 | 1.000 | 0.000 | 0.133 | 0.00 | 1.000 |
| condition index | -0.425 | 0.135 | -3.15 | **0.003** | 0.113 | 0.133 | 0.85 | 0.397 |
|  | **agglutination titer** | | | | | | | |
| Intercept | 0.000 | 0.150 | 0.000 | 1.000 | 0.000 | 0.111 | 0.00 | 1.000 |
| condition index | 0.017 | 0.166 | 0.099 | 0.921 | -0.498 | 0.112 | -4.44 | **0.000** |
| cloacal microbiome | -0.082 | 0.166 | -0.493 | 0.624 | 0.327 | 0.112 | 2.91 | **0.005** |
|  | immunity index | | | | | | | |
| Intercept | 0.000 | 0.138 | 0.000 | 1.000 | 0.000 | 0.102 | 0.000 | 1.000 |
| condition index | 0.167 | 0.152 | 1.10 | 0.278 | -0.066 | 0.103 | -0.641 | 0.524 |
| cloacal microbiome | -0.302 | 0.152 | -1.98 | 0.054 | -0.643 | 0.103 | -6.26 | **0.000** |
|  | **eggshell microbiome** | | | | | | | |
| Intercept | 0.000 | 0.117 | 0.00 | 1.000 | 0.000 | 0.128 | 0.00 | 1.000 |
| cloacal microbiome | -0.619 | 0.117 | -5.28 | **0.000** | 0.290 | 0.128 | 2.26 | **0.027** |
|  | **yolk [IgY]** | | | | | | | |
| Intercept | 0.000 | 0.083 | 0.00 | 1.000 | 0.000 | 0.128 | 0.00 | 1.000 |
| agglutination titer | -0.222 | 0.087 | -2.54 | **0.015** | -0.043 | 0.130 | -0.33 | 0.742 |
| immunity index | 0.789 | 0.085 | 9.26 | **0.000** | -0.302 | 0.137 | -2.20 | **0.032** |
| eggshell microbiome | -0.359 | 0.086 | -4.16 | **0.000** | -0.285 | 0.136 | -2.10 | **0.040** |
|  | **albumen pH** | | | | | | | |
| Intercept | 0.000 | 0.136 | 0.00 | 1.000 | 0.000 | 0.134 | 0.00 | 1.000 |
| agglutination titer | 0.131 | 0.142 | 0.92 | 0.361 | -0.025 | 0.136 | -0.18 | 0.855 |
| immunity index | -0.148 | 0.139 | -1.06 | 0.293 | 0.127 | 0.144 | 0.88 | 0.381 |
| eggshell microbiome | 0.405 | 0.141 | 2.88 | **0.006** | -0.088 | 0.142 | -0.62 | 0.539 |
|  | **albumen [lysozyme]** | | | | | | | |
| Intercept | 0.000 | 0.149 | 0.000 | 1.000 | 0.000 | 0.126 | 0.00 | 1.000 |
| agglutination titer | -0.161 | 0.157 | -1.03 | 0.308 | 0.213 | 0.128 | 1.67 | 0.101 |
| immunity index | -0.201 | 0.153 | -1.31 | 0.196 | 0.297 | 0.136 | 2.18 | **0.034** |
| eggshell microbiome | 0.004 | 0.168 | 0.02 | 0.983 | 0.202 | 0.134 | 1.51 | 0.138 |
| albumen pH | -0.014 | 0.166 | -0.09 | 0.932 | -0.095 | 0.128 | -0.74 | 0.460 |

* bold values denote statistically significant associations (critical *P* = 0.05)

**Table S3.** Summary of bootstrap t-test for experimental effect on Partial Least Squares path coefficients

| Path from | To | Path  coefficient global | Path coefficient  high diversity | Path coefficient  low diversity | Absolute difference  high-low diversity | t | df | *P* | FDR *q*-value* |
| --- | --- | --- | --- | --- | --- | --- | --- | --- | --- |
| condition index | cloacal microbiome | 0.091 | -0.425 | 0.113 | 0.539 | 2.91 | 103 | 0.002 | **0.030** |
| condition index | agglutination titer | -0.323 | 0.017 | -0.498 | 0.514 | 2.47 | 103 | 0.008 | **0.030** |
| condition index | immunity index | -0.006 | 0.167 | -0.066 | 0.233 | 0.50 | 103 | 0.311 | 0.380 |
| cloacal microbiome | agglutination titer | 0.223 | -0.082 | 0.327 | 0.408 | 2.13 | 103 | 0.018 | **0.057** |
| cloacal microbiome | immunity index | 0.283 | -0.302 | -0.643 | 0.340 | 0.65 | 103 | 0.257 | 0.374 |
| cloacal microbiome | eggshell microbiome | 0.121 | -0.619 | 0.290 | 0.908 | 0.52 | 103 | 0.302 | 0.380 |
| agglutination titer | yolk [IgY] | 0.010 | -0.222 | -0.043 | 0.179 | 0.91 | 103 | 0.181 | 0.374 |
| agglutination titer | albumen pH | 0.054 | 0.131 | -0.025 | 0.156 | 0.29 | 103 | 0.385 | 0.385 |
| agglutination titer | albumen [lysozyme] | 0.064 | -0.161 | 0.213 | 0.374 | 2.54 | 103 | 0.006 | **0.030** |
| immunity index | yolk [IgY] | 0.702 | 0.789 | -0.302 | 1.091 | 2.57 | 103 | 0.006 | **0.030** |
| immunity index | albumen pH | -0.147 | -0.148 | 0.127 | 0.275 | 0.81 | 103 | 0.211 | 0.374 |
| immunity index | albumen [lysozyme] | -0.160 | -0.202 | 0.297 | 0.498 | 1.04 | 103 | 0.149 | 0.374 |
| eggshell microbiome | yolk [IgY] | 0.015 | -0.359 | -0.285 | 0.074 | 0.88 | 103 | 0.191 | 0.374 |
| eggshell microbiome | albumen pH | -0.254 | 0.405 | -0.088 | 0.493 | 0.72 | 103 | 0.238 | 0.374 |
| eggshell microbiome | albumen [lysozyme] | 0.125 | 0.004 | 0.202 | 0.198 | 0.37 | 103 | 0.356 | 0.380 |
| albumen pH | albumen [lysozyme] | -0.045 | -0.014 | -0.095 | 0.081 | 0.41 | 103 | 0.341 | 0.380 |

* bold values denote significant differences (critical FDR *q* = 0.1)

REFERENCES

van Veelen, H. P. J., Falcão Salles, J., Matson, K. D., van der Velde, M., & Tieleman, B. I. (2020). Microbial environment shapes immune function and cloacal microbiota dynamics in zebra finches Taeniopygia guttata. *Animal Microbiome*, *2*(1), 21. doi: 10.1186/s42523-020-00039-3
